# Trickle infection with *Heligmosomoides polygyrus* results in decreased worm burdens but increased intestinal inflammation and scarring

**DOI:** 10.3389/fimmu.2022.1020056

**Published:** 2022-12-08

**Authors:** Anupama Ariyaratne, Sang Yong Kim, Stephen M. J. Pollo, Shashini Perera, Hongrui Liu, William N. T. Nguyen, Aralia Leon Coria, Mayara de Cassia Luzzi, Joel Bowron, Edina K. Szabo, Kamala D. Patel, James D. Wasmuth, Meera G. Nair, Constance A. M. Finney

**Affiliations:** ^1^ Department of Biological Sciences, Faculty of Science, University of Calgary, Calgary, AB, Canada; ^2^ Host Parasite Interactions Training Network, University of Calgary, Calgary, AB, Canada; ^3^ Division of Biomedical Sciences, School of Medicine, University of California Riverside, Riverside, CA, United States; ^4^ Faculty of Veterinary Medicine, University of Calgary, Calgary, AB, Canada; ^5^ Departments of Physiology and Pharmacology, Faculty of Medicine, University of Calgary, Calgary, AB, Canada

**Keywords:** helminth, granuloma, trickle infection, ADAMTS, intestinal parasite, tissue scarring

## Abstract

**Introduction:**

Intestinal roundworms cause chronic debilitating disease in animals, including humans. Traditional experimental models of these types of infection use a large single-dose infection. However, in natural settings, hosts are exposed to parasites on a regular basis and when mice are exposed to frequent, smaller doses of *Heligmosomoides polygyrus*, the parasites are cleared more quickly. Whether this more effective host response has any negative consequences for the host is not known.

**Results:**

Using a trickle model of infection, we found that worm clearance was associated with known resistance-related host responses: increased granuloma and tuft cell numbers, increased levels of granuloma IgG and decreased intestinal transit time, as well as higher serum IgE levels. However, we found that the improved worm clearance was also associated with an inflammatory phenotype in and around the granuloma, increased smooth muscle hypertrophy/hyperplasia, and elevated levels of *Adamts* gene expression.

**Discussion:**

To our knowledge, we are the first to identify the involvement of this protein family of matrix metalloproteinases (MMPs) in host responses to helminth infections. Our results highlight the delicate balance between parasite clearance and host tissue damage, which both contribute to host pathology. When continually exposed to parasitic worms, improved clearance comes at a cost.

## Introduction

Gastrointestinal parasitic nematodes often cause chronic recurring infections. Hosts mount a strong immune response to nematode parasites, essential to control worm burden as well as host tissue damage. While many hosts are infected and unable to clear their infection, they can limit excessively damaging worm burdens, implying immune regulatory mechanisms are at play (1). The balance and efficacy of the response is dependent on parasite killing and wound healing mechanisms that act in concert to maximize host fitness.

*Heligmosomoides polygyrus* is an enteric nematode parasite of mice (2, 3). Ingested larvae encyst in the host intestinal wall and mature into adults that escape into the lumen. This process takes approximately one week, after which adults remain in the intestinal lumen for the duration of infection. *H. polygyrus* tissue-dwelling stages cause the release of alarmins from epithelial cells (including tuft cells) as they damage the intestinal wall (1, 4, 5). Alarmin-activated innate lymphoid cells and Th2 polarized CD4^+^ T cells produce Th2 cytokines (6–9) which promote innate immune cell influx to the intestine (3, 6, 10). The accumulation of immune cells is referred to as a granuloma (7, 11, 12) and an increase in granuloma size and number is associated with increased resistance to *H. polygyrus* (13).

Granulomas can be categorized as early-stage, containing developing worms, and late-stage, where worms have escaped into the intestinal lumen, and/or been killed. Within the early-stage granuloma, the immune response must balance the dual demands of incapacitating or killing the developing nematode, while also healing the damage caused by the growing worm. In late-stage granulomas, the immune response has shifted to focus on healing [reviewed in (14)]. Eliminating the tissue stage parasites relies on antibody-dependent cell-mediated cytotoxicity (ADCC) by macrophages and eosinophils (7, 15, 16), the main cellular players within the granuloma. AAMs and eosinophils produce immunoregulatory and wound healing molecules (17, 18) such as Ym1, RELM-α (3, 19, 20), and Arginase 1 (9, 21), which promote extracellular matrix (ECM) deposition during helminth infections (22–25). This healing process has been associated with fibrosis and scarring during chronic helminth infection through the excessive deposition of collagen, a major component of the ECM (26). How the host balances parasite clearance and effective tissue remodeling within the granuloma is not fully understood.

Immune responses are not only generated to tissue dwelling parasitic stages but also to the adult worms found in the intestinal lumen. The cytokines IL-4 and IL-13 enhance smooth muscle contractility of the intestine *via* STAT6 dependent pathways (27) to help eliminate adult worms (28–30). IL-4, IL-9, and IL-13 regulate goblet cell hyperplasia and increase mucus production during gastrointestinal (GI) nematode infections (31, 32) which presumably makes it more difficult for adult parasites to coil around intestinal villi. *H. polygyrus* infections also induce polyclonal and parasite-specific antibody responses, which function to damage worm larvae as they develop and limit adult female egg production (1, 33–35).

Most of the murine studies on helminth infection use a bolus model of infection (one large dose), with some groups adopting a drug clearance model (bolus infection, drug clearance, bolus infection) to simulate mass drug administration programs in human populations (7, 33). However, under natural conditions, gastrointestinal nematodes are ubiquitous in the environment (36) and hosts are constantly encountering them. Hence, we and others have set up experimental infection models using trickle infections to study host-nematode infections in a more natural setting (37–41). We use multiple low doses of larvae, given over a specific time period to achieve this.

*H. polygyrus* trickle infections in genetically resistant and susceptible strains of mice reveal that the frequency of infection is an important determinant of parasite expulsion, where frequently infected mice eliminate worms more rapidly than mice infected with the same total number of larvae but in less frequent doses (40). Others have implicated improved antibody and innate immune cell responses to tissue dwelling parasites as key elements for the reduced worm burdens observed during trickle infections (37, 40, 41). In our model, improved clearance was associated with known resistance-related host responses: increased granuloma and tuft cell numbers, increased levels of granuloma IgG, decreased intestinal transit time and higher serum IgE levels. However, we found that the improved worm clearance observed in trickle-infected animals was also associated with an inflammatory phenotype in and around the granuloma, increased smooth muscle hypertrophy/hyperplasia and elevated *Adamts* gene expression. Many studies have focused on understanding the mechanisms involved in damaging/killing worms, but fewer have focused on the wound healing processes involved in creating and resorbing granulomas. This is key, as there is a delicate balance between parasite clearance and host pathology which ultimately impact host fitness.

## Materials and methods

### Mice, parasites, and antigen

Female and male C57Bl/6 and BALB/c mice aged 6-8 weeks (bred and maintained at the animal care facility, Department of Biological Sciences, University of Calgary, Canada or University of California, Riverside, USA) were used. All animal experiments were approved by the University of Calgary’s Life and Environmental Sciences Animal Care Committee (protocol AC17-0083) and the University of California, Riverside’s Institutional Animal Care and Use Committee (https://or.ucr.edu/ori/committees/iacuc.aspx; protocol A-20180023). All protocols for animal use and euthanasia were in accordance with either the Canadian Council for Animal Care (Canada) or National Institutes of Health (USA) guidelines. Animal studies are in accordance with the provisions established by the Animal Welfare Act and the Public Health Services (PHS) Policy on the Humane Care and Use of Laboratory Animals.

**Figure 1:**
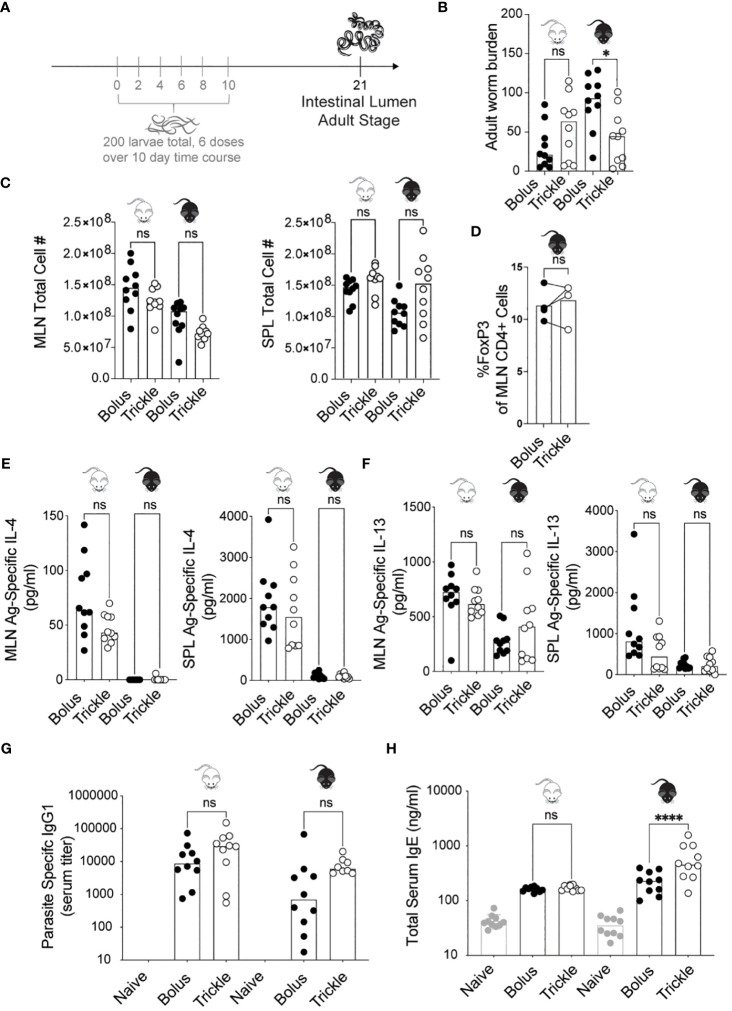
The reduced worm burden in trickle-infected C57Bl/6 mice is associated with elevated serum IgE. 6-8 week old C57Bl/6 and BALB/c mice were infected with 200 *H polygyrus* larvae according to the bolus and trickle infection regimes. **(A)** Trickle infection regime: mice are infected with 200 larvae in total, but in multiple doses over the course of infection (in grey). Doses of ~33 larvae are trickled on days 0, 2, 4, 6, 8 and 10 post-infection. There is a 10-day window after the final dose to allow parasites to fully develop into adults and migrate from the intestinal tissue to the intestinal lumen. **(B)** Adult worms were counted in the small intestine using a dissection microscope. **(C)** Single cell suspensions were isolated from the mesenteric lymph nodes and spleens. Viable cell numbers in the MLN (left) and SPL (right). Single cell suspensions were either used for flow cytometry or cultured for 48 hours in the presence of *H polygyrus* antigen. **(D)** Percentage of Foxp3^+^ cells within the CD4^+^ population of MLN cells were measured by flow cytometry. **(E)** IL-4 (MLN and SPL) and **(F)** IL-13 (MLN and SPL) cytokine levels were measured in the supernatant by ELISA. Serum antibody levels were measured by ELISA for **(G)** parasite specific IgG1 and **(H)** total IgE. Levels in naïve controls were undetectable for parasite specific IgG1. For all panels except D, graphs represent pooled data from 2 experiments, bars represent the median, with a minimum of 3 mice per group per experiment. For panel D, each circle represents one experiment using cells pooled from 5 mice. BALB/c mice (white mouse) and C57Bl/6 mice (black mouse) were infected according to the bolus (black circles) and trickle (white circles) regimes. A normality test was performed (Anderson-Darling) followed by a Kruskal Wallis test with Dunn’s multiple comparisons test to test for statistical significance between trickle and bolus groups; for panel **(D)**, a paired T-test was performed; n.s., not significant; *p<0.05, ****p<0.0001.

Infected mice were orally gavaged with 200 third stage *Heligmosomoides polygyrus* larvae (maintained in house, stock was a gift from Dr. Allen Shostak, University of Alberta, Canada and Dr Lisa Reynolds,University of Victoria, Canada) and euthanized at either 7 (D7), 14 (D14) or 21 (D21) days post initial infection. Mice were infected according to the bolus or trickle infection regimes (**Figure 1A** and **Supplementary Figure 1A**). To avoid differences in counts during the trickle infections, on day 0, two identical solutions were made up (200 worms/100ul). One was used to infect the bolus infected mice on day 0 and one was used for the trickle-infected mice. The solution for the trickle-infected animals was divided into six equal parts. Each part was made up to 100ul using water. Animals were gavaged with a diluted solution on days 0, 2, 4, 6, 8, 10. Using the 6 doses, trickle-infected animals received 200 larvae in total over 10 days.

*H. polygyrus* antigen was prepared by collecting live adult worms from 14-day infected mice using modified Baerman’s apparatus. Worms were washed multiple times and homogenized in PBS using a glass homogenizer. The resulting solution was centrifuged (13, 000 g, 10 minutes, 4°C) and the supernatant filtered (0.2μm filter, Nalgene). The protein concentration was calculated using the Bradford assay. The antigen was stored at 15 mg/ml at -80°C.

### Adult worm burden and granuloma number

Small intestines of infected mice were harvested and opened longitudinally. The number of adult worms present in the intestinal lumen and of granulomas present along the length of the small intestine were counted using a dissection microscope.

### Transit time

Gastrointestinal transit time was measured one day prior to euthanasia. Mice were fasted for 6 hours and 200 μl of 5% Evans blue (Sigma) in 5% gum arabic (ACROS organics) was orally gavaged using a ball tip 20 gauge 1.5’’, 2.25mm curved animal feeding needle. Each mouse was labelled, with the time of dye administration recorded. Mice were transferred to clean empty cages and the time to pass the first blue fecal pellet was recorded. Gastrointestinal transit time was calculated for each mouse.

### Cell isolation and *in vitro* re-stimulation assay

Mesenteric lymph nodes (MLN) and spleens (SPL) were mechanically dissociated into single cell suspensions. Cells were counted using a Beckman-Coulter ViCell XR. MLN and SPL were cultured at 1 x 10^6^ cells/ml for 48 hours in RPMI medium, 10% FCS, 1% L-glutamine, 1% penicillin/streptomycin (supplemented RPMI 1640) in the presence of 10 μg/ml *H. polygyrus* antigen or 2 μg/ml concanavalin A (Sigma) at 37^0^C with 5% CO_2_. Supernatants were collected for cytokine measurements. Measurements for antigen-specific production were not included in the analysis unless cytokine production was observed in the wells with concanavalin A stimulation.

### Flow cytometry

MLN single cell suspensions were stained and analyzed according to (42). Cells were stained for: viability (BV510), CD4 (BUV395), and Foxp3 (BV421), all purchased from BD Biosciences, Canada. Cells were blocked with rat α-mouse CD16/32 from Biolegend, USA, surface stained for CD4 and stained intracellularly for Foxp3. Cells were run on an LSRFortessa X-20 flow cytometer and data were analyzed using FlowJo software (v10 & 7.6.5, FlowJo LLC, USA). For analysis, doublets and dead cells were removed from the analysis.

### Serum

Blood samples were collected using a terminal cardiac bleed. Blood was left to clot for 30 minutes and then centrifuged twice at 11, 000 g at 4°C for 10 minutes. Serum was collected and used either fresh or stored at -80°C.

### Intestinal tissue homogenates

Small intestines were opened longitudinally and washed with PBS to remove luminal content. The mucosal surface was identified under a dissecting microscope. The mucosal surface (with its mucus) was gently scraped using a glass slide. Scrapings were weighed, added to 500 μl lysis buffer (10 μM tris HCl, 0.025% sodium azide, 1% tween 80, 0.02% phenylmethylsulfonyl fluoride) with one complete protease inhibitor tablet (Roche diagnostics GmbH, Germany) and homogenized using a bead beater (40 seconds at speed 6 using the Fast-prep-24 bead beater, MP biomedical). The homogenate was centrifuged at 11, 000 g at 4°C for one hour. Supernatants were collected and used fresh or stored at -80°C.

### ELISAs

Cytokines in serum and intestinal tissue homogenates were measured by ELISA according to manufacturer’s guidelines (R & D systems, DY404 kit for IL-4, DY413 kit for IL-13, DY594 kit for IL-21). Total IgE (BD, 555248) and IgA (capture antibody, BD, 556969, detection antibody, BD, 556978) levels were measured by ELISA according to manufacturer’s instructions.

Antigen specific antibody responses were also measured by ELISA. ELISA microplates were coated with 10 μg/ml *H. polygyrus* antigen in carbonate buffer (0.1mM NaHCO_3_, pH 9.6), overnight at 4°C. Plates were blocked with 2% BSA in TBS/0.05% tween 20 for 2 hours at 37°C. Sera were diluted in TBS Tween and added to wells overnight at 4°C. Antigen- specific IgG_1_ was detected with HRP-conjugated corresponding detection antibodies (anti-IgG_1_ (BD, 553441) with TMB peroxidase substrate (T3405, Sigma). The reaction was stopped using 1M H_2_SO_4_ solution and the color change was read at 450nm.

### Histology

Consecutive formalin fixed paraffin embedded mouse small intestinal sections were stained with H & E by the University of Calgary’s Veterinary Diagnostic Services Unit. For cell identification (eosinophils and macrophages), one photograph of each granuloma was taken at x400 magnification using an Olympus BX 34 microscope and the Olympus CellSens software. Cells were identified by their morphology and staining patterns.

For immunofluorescence, the sections were stained with either anti-mouse IgG1 (BD 553443), anti-mouse DCLK1 (AB31704), anti-mouse ADAMTS4 (BD, 559354) or their respective isotypes overnight at 4°C. Slides were mounted with FluoroshieldTM with DAPI (Sigma-Aldrich F6057). Images were acquired using the Zeiss Axio ZoomV.16 stereoscopic microscope (Bio Core Facility, Department of Biological Sciences, University of Calgary) or the Thorlabs Tide whole-slide scanning microscope (Live cell imaging facility, Snyder institute of chronic diseases). Brightfield and fluorescence images were taken either under the 2.3x objective with the AxioCam High Resolution color (HRc) and High-Resolution mono (HRm) cameras, or the 20x/0.75 NA objective of the slidescanner respectively. Zeiss Zen (blue edition) software or Thorlabs Tide LS image acquisition software was used. DAPI and FITC fluorophores were used with excitation wavelengths at 359 and 488nm, respectively. Brightness and contrast were adjusted in photoshop.

For Second Harmonic Generation (SHG) Imaging, all samples were imaged using a Thorlabs Bergamo Multiphoton Microscopy Platform (Thorlabs, New Jersey, USA) with a Olympus XLPLan 25X/1.05NA water immersion objective. A Chameleon Ti: Sapphire two-photon laser (Coherent, California, USA) tuned to 900nm was used to collect SHG signal *via* a PMT with a 447/60 bandpass filter. To ensure consistency and good imaging quality of collagen fibers, laser power and gain were optimized and remained the same throughout all samples imaged. Images from each granuloma were collected in z-stacks of 16 images taken at 1µm intervals. Z-stacks were processed in FIJI (ImageJ version 1.53s) (43) where maximum intensity projections were created analyzed for average pixel intensity.

### Nanostring nCounter gene expression assay

Intestinal tissue from naïve mice or dissected pooled granulomas from infected mice were snap frozen in liquid nitrogen and RNA was isolated using phenol-chloroform extraction (TRIZOL, Sigma). RNA was quantified using a nanodrop and 50 ng was used for the Myeloid Innate Immunity V2 panel (NanoString) according to the manufacturer’s guidelines. Gene expression analysis was conducted in R (44). Gene counts obtained *via* the NanoString hybridization assay were normalized with NanostringNorm (45) using the negative control probes, positive control probes and housekeeping genes Eif2b4, Polr1b, and Edc3. Of the 20 housekeeping genes included in the assay, only Eif2b4, Polr1b, and Edc3 were found to have consistent expression among all samples in preliminary comparisons. Therefore Eif2b4, Polr1b, and Edc3 were the only housekeeping genes used for normalization in subsequent analyses. The normalized counts were then compared using DESeq2 (46) to find differentially expressed genes in pairwise comparisons between treatment groups. A false discovery rate adjusted p-value cut-off of 0.05 and a fold-change cutoff of two were used to identify genes that were differentially expressed in each pairwise comparison. The data discussed in this publication have been deposited in NCBI’s Gene Expression Omnibus (47) and are accessible through GEO (Gene Expression Omnibus) Series accession number GSE164319. Male mice were used for D21 and naive while female mice were used for D7 experiments. Low coverage sequencing of early granulomas indicates no difference in myeloid genes between male and female mice (data not shown).

### Statistical analysis (except for nanostring results)

Linearity was assessed using an Anderson Darling test. For nonparametric data, Mann Whitney/Kruskal Wallis tests with Dunn’s multiple comparisons were used to assess differences between either two or more experimental groups using GraphPad Prism. For parametric data, we used T-tests/ANOVA with Sidak’s multiple comparisons.

## Results

### Increased worm clearance in trickle-infected animals is associated with increased levels of serum IgE, but no other changes in key systemic cytokine or antibody responses

Administering *H. polygyrus* larvae in low frequent doses to C57Bl/6 mice changed their susceptibility to infection. Two inbred strains of mice, C57Bl/6 (genetically susceptible) and BALB/c (genetically resilient) (13) were infected with *H. polygyrus* according to the bolus (single 200 larvae dose) or trickle infection regimes (200 larvae dose given over the course of 10 days, **Figure 1A**). When given a bolus infection of 200 worms, BALB/c mice, being partially resistant to *H. polygyrus*, eliminated most of their worms by 21 days post-infection (D21, mean of 31, SD +/- 27, **Figure 1B**). In contrast, C57Bl/6 mice, being susceptible to *H. polygyrus*, harboured high numbers of adult worms (mean of 88, SD +/- 35, **Figure 1B**). When infected according to the trickle protocol however, C57Bl/6 mice also eliminated most of the adult worms by 21 days post infection (mean of 44, SD +/- 34, **Figure 1B**). This difference in worm burden was already apparent at D14 (**Supplementary Figure 1B**), although many worms in the trickle-infected animals were still developing in the tissue at D14 (**Supplementary Figure 1A**), skewing the results at this time point.

*H. polygyrus* clearance has been associated with a strong Th2 response, specifically increases in IL-4 and IL-13 cytokines (32, 48, 49). As the Th2 immune response develops in response to *H. polygyrus*, MLN (mesenteric lymph nodes) and SPL (spleen) cell numbers increase (50). We found no differences in cell number between the trickle- and bolus-infected groups in either of the organs (**Figure 1C**). At D14, we observed increased MLN cell numbers in the bolus compared to trickle-infected group (**Supplementary Figure 1C**). Tregs have been shown to modulate *H. polygyrus* infection outcome (51), yet we saw no changes in the proportion of Tregs (defined as Foxp3^+^CD4^+^ cells) between the trickle and bolus-infected groups at either D14 or D21 (11-16% Foxp3^+^ cells of CD4^+^ cells, **Supplementary Figure 1D** and **Figure 1D**). We measured the levels of the antigen-specific Th2 cytokine production (IL-4 and IL-13) in both the MLN and SPL as well as in the serum of mice by ELISA (**Figures 1E, F**). Levels of IL-4 measured in BALB/c mice at D21 post infection were higher than in C57Bl/6 mice (**Figure 1E**), as has previously been reported (13). No differences were found in cytokine levels between trickle and bolus-infected C57Bl/6 mice at D21 post-infection. Levels of IL-4 and IL-13 were undetectable in the serum, confirming the findings of others (3). In C57Bl/6 animals, we further measured the levels of four cytokines implicated in immune responses against helminths [IFNγ, IL-10, IL-9 and IL-5 (3, 52, 53)] in the MLN and SPL. No difference in these were observed between the trickle and bolus-infected animals (data not shown).

Parasite specific IgG1 and total IgE both increase during primary and secondary *H. polygyrus* infection (33). IgG1 has been associated with parasite clearance (1) while IgE is thought to reduce parasite fecundity (33). We measured an increase in parasite specific IgG1 by D21 post-infection, as has previously been reported (33). No differences were observed between trickle and bolus-infected animals (**Figure 1G**). No levels of *H. polygyrus* antigen-specific IgE, IgG2c or IgA were detectable in the serum of infected mice at any post-infection time point; both larval and adult parasite antigen were tested. However, an increase in total serum IgE was measured in both mouse strains (**Figure 1H**), with increased levels in the trickle-infected C57Bl/6 animals.

### In the small intestine, increased worm clearance in trickle-infected animals is associated with increased tuft cell number

**Figure 2:**
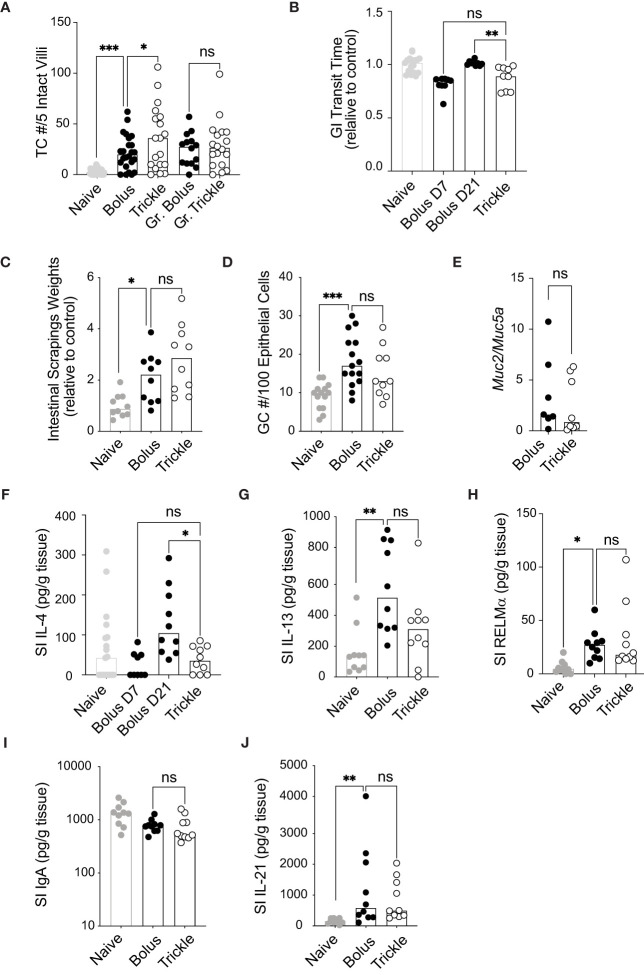
The reduced worm burden in trickle-infected C57Bl/6 mice is associated with increased tuft cell numbers. 6-8 week old C57Bl/6 mice were infected with 200 *H polygyrus* larvae according to the bolus and trickle infection regimes. **(A)** Paraffin embedded swiss rolls were stained with anti-DCLK1. The number of tuft cells were counted in 5 intact villi within the small intestine. Photographs from ten different areas of the small intestine were counted per animal (a minimum of five from areas with no granulomas and a minimum of three from areas with granulomas - Gr). **(B)** Mice were fasted for 6 hours followed by Evans Blue administration. Time from dye administration to the passing of dyed fecal pellets was measured and normalised to control animals. **(C)** Small intestines were dissected and scraped using a glass slide leaving only the serosa. The intestinal scrapings were weighed for each mouse and normalised to control animals. **(D)** Paraffin embedded swiss rolls were stained with Alcian blue. The average number of goblet cells in 5 intact and continuous villi within the small intestine was collected and normalised to the epithelial cell number. **(E)** RNA was extracted from whole intestinal tissue and *Muc2* and *Muc5a* expression were measured normalised to the β-actin housekeeping gene. **(F-J)** IL-4, IL-13, RELMα, total IgA and IL-21 were measured in the intestinal scrapings by ELISA. Naive mice (grey circles) and mice infected according to the bolus (black circles) and trickle (white circles) regimes. Graphs represent pooled data from 2 experiments, bars represent the median, with a minimum of 2 mice per group per experiment. A normality test was performed (Anderson-Darling) followed by Kruskal Wallis tests with Dunn’s multiple comparisons test to test for statistical significance between trickle and bolus groups, n.s., not significant, *p<0.05, **p<0.01, ***p<0.001.

Worm infections result in physiological changes in the small intestine that have been linked to promoting worm expulsion. These include increased tuft cell numbers (54), increased intestinal smooth muscle contractility (55) (and therefore, decreased intestinal transit time) as well as increased mucus production (3). We saw a significant increase in tuft cell numbers three weeks after infection as expected (54) (**Figure 2A**), from approximately 3 (SD +/- 2.7) cells in naïve animal to more than 22 cells per 5 villi in infected animals. In regions which were not in proximity to granulomas, we also found that trickle-infected animals have a 50% increase in tuft cell number (36, SD +/- 31 *vs*. 22, SD +/- 17). In areas around the granulomas, this difference was not observed, in accordance with recent data showing that *H. polygyrus* can inhibit tuft cell expansion (56). Transit time, measured by the time to pass dyed gavaged material, was reduced in D7 bolus-infected (by 18%, SD +/- 7.5) and D21 trickle-infected (by 12%, SD +/- 11) C57Bl/6 animals but not in D21 bolus-infected animals (0%, SD +/- 2.3, **Figure 2B**). This suggests transit time decreases transiently early in infection. Mucus production, measured indirectly through intestinal tissue weights and goblet cell number, increased with infection but did not differ between the infected groups (**Figures 2C, D**). The ratio of *Muc2/Muc5a* gene expression, which increased in trickle-infected *Trichuris*-infected animals (57), was no different between the *H. polygyrus* bolus and trickle-infected groups (**Figure 2E**).

We then measured the levels of the Th2 cytokines IL-4 and IL-13 by ELISA in intestinal tissue. They have been associated with stimulating increased mucus production in the small intestine (58). Despite no differences in goblet cell numbers and intestinal weight, IL-4 levels were significantly reduced in trickle compared to bolus-infected animals at D21 (**Figure 2F**). Levels between D7 bolus-infected and D21 trickle-infected animals were similar, indicating that IL-4 levels are time/dose dependent. IL-13 and RELMα levels, by contrast were increased compared to naïve animals but remained similar between trickle and bolus-infected groups (**Figures 2G, H**). Levels of IFNγ, IL-5, IL-9 and IL-10 were below the detection limit of the assay for intestinal tissue for all mice tested.

Mucosal IgA levels, regulated by the cytokine IL-21 (59), are also increased in the presence of intestinal dwelling parasites (60). Intestinal IgA levels did not differ between bolus or trickle-infected mice at D21 post-infection (**Figure 2I**). IL-21 levels were increased at this time point, from 98 pg/ml, SD +/- 84 in naïve animals to approximately 950 pg/ml in infected animals (**Figure 2J**). However, like IgA levels, they were not different between trickle- and bolus-infected animals.

### Increased worm clearance in trickle-infected animals is associated with increased granuloma number, size and IgG levels as well as increased smooth muscle hypertrophy/hyperplasia

**Figure 3:**
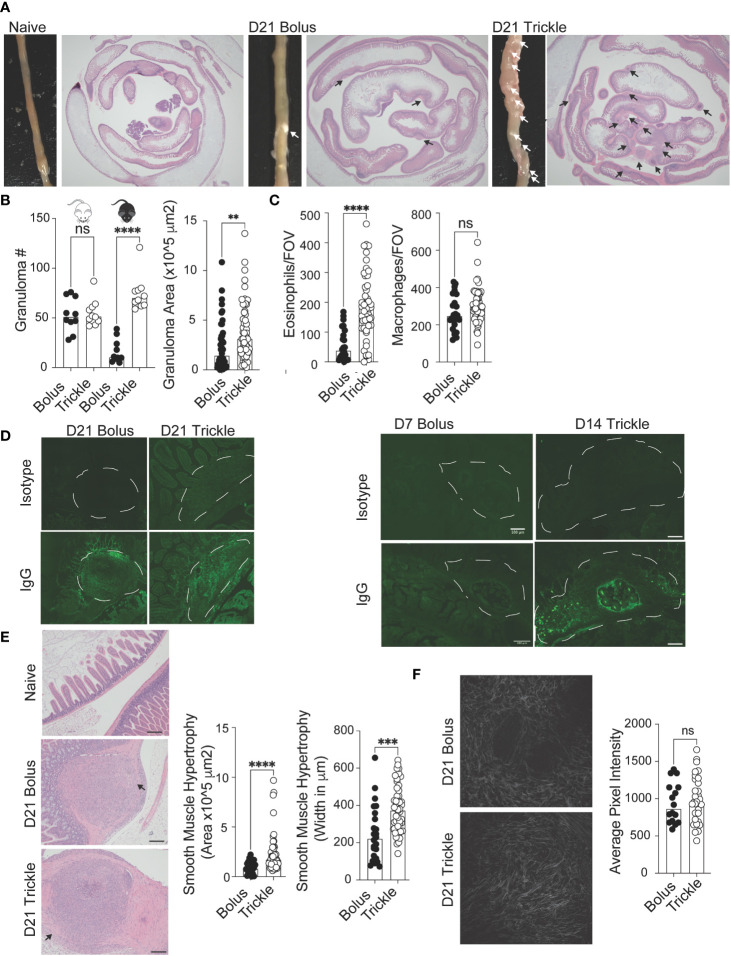
The reduced worm burden in trickle-infected C57Bl/6 mice is associated with increased granuloma size, number and level of IgG as well as intestinal muscle hypertrophy/hyperplasia. 6-8 week old C57Bl/6 and BALB/c mice were infected with 200 *H polygyrus* larvae according to the bolus and trickle infection regimes. **(A)** Representative images of granuloma pathology. Representative photographs showing the small intestine of naïve, D21 bolus and D21 trickle-infected C57Bl/6 mice. White arrows point to granulomas. Whole small intestinal swiss rolls were cut into 6 µm paraffin embedded sections and stained with H & E. Representative photographs highlight the granulomas (black arrows). **(B)** Granulomas were counted on the outside of the small intestine using a dissection microscope for identification (left). Granuloma area was measured on H & E sections (right). **(C)** Eosinophil (left) and macrophage (right) counts within the center of the granulomas. H & E slides were used to identify eosinophils and macrophages at x400 magnification. Cells were counted for one field of view (FOV) per granuloma. **(D)** Formalin fixed, paraffin-embedded 6 μm sections were obtained from small intestine swiss rolls. These sections were co-stained with anti-mouse IgG1 and DAPI. Whole sections were studied using the Thorlabs Tide whole-slide scanning microscope (20x objective). Representative granulomas (white dashed line) from D21 (left, no developing worms) and D7 bolus/D14 trickle (right, developing worms present) infected animals with both isotype control (top) and antibody stain (bottom). **(E)** Left: representative images of smooth muscle cell hypertrophy/hyperplasia on H & E-stained sections. Pink areas around granulomas represent smooth muscle. Black arrows point to areas where collagen density was measured, scale bar = 200um. Right: intestinal smooth muscle area and width were measured on H & E slides for each granuloma **(F)** Left: representative photographs of collagen within the granulomas using SHG imaging. Right: Collagen density per FOV measured using ImageJ. Data pooled from 2 experiments with a minimum of 2 mice per group per experiment. A normality test was performed (Anderson-Darling) followed by Kruskal Wallis tests with Dunn’s multiple comparisons test and/or an unpaired T-test/Mann-Whitney test to test for statistical significance between trickle and bolus groups, n.s., not significant, **p<0.01, ***p<0.001, ****p<0.0001.

Granulomas are a characteristic response to intestinal roundworms and are identifiable by eye (**Figure 3A**) (13). Bolus-infected BALB/c mice had higher granuloma counts at D21 post-infection (53, SD +/- 17) compared to C57Bl/6 bolus-infected animals (17, SD +/- 12, **Figure 3B**, left). However, in trickle-infected C57Bl/6 mice, granuloma numbers, like worm burdens, were similar to BALB/c mice (75, SD +/- 18, **Figure 3B**, left). In C57Bl/6 mice, trickle-infected animals also had larger granulomas (**Figure 3B**, right) which contained a greater number of eosinophils (**Figure 3C**, left) but similar numbers of macrophages (**Figure 3C**, right) compared to bolus-infected animals. To study worm killing mechanisms in the granuloma, we measured levels of IgG by immunofluorescence. At D21 post-infection, no obvious differences in staining were obtained (**Figure 3D**, left). This is not surprising since at this time point, developing worms have exited the tissue. We also stained for IgG in parasite-containing granulomas from the bolus-infected animals (at D7 when these can be observed) as well as trickle-infected mice (at D14, when these can be observed, **Figure 3D**). Here, we found a lack of IgG in the granulomas from D7 animals (also undetectable in the serum) compared to strong IgG staining around the developing worms in the granulomas of trickle-infected animals (**Figure 3D**, right).

Worm clearance is associated with elevated Th2 responses, which, in a chronic setting, can lead to fibrosis (61). Since intestinal nematodes cause considerable damage to the intestinal mucosa through the creation of granulomas, we wanted to know whether the healing of these structures was affected by the infection type. Using H & E slides (**Figure 3E**, left), we measured histological abnormalities associated with the granulomas. This included the area and maximum width of smooth muscle (**Figure 3E**) as well as collagen deposition (**Figure 3F**) (62). Smooth muscle hypertrophy/hyperplasia was increased in the trickle-infected animals. Although trickle-infected granulomas are also increased in size, we found no correlation between the measured area of the granuloma *vs*. that of the smooth muscle (R^2^ = 0.15, data not shown). Fibrosis/Scarring is associated with collagen deposition (14). Using second harmonic generation (SHG) imaging, we assessed the density of collagen in the granulomas (**Figure 3F**, left). We found no difference between the groups in collagen deposition per field of view (FOV, **Figure 3F**, right).

### Increased worm clearance in trickle-infected animals is associated with a unique gene expression pattern in the granulomas linked to inflammation and tissue scarring

We isolated all granulomas along the small intestine of bolus and trickle-infected C57Bl/6 mice at 21 days post-infection. These are late-stage granulomas, collected 10 days after the last trickle dose, at a point when all worms have developed into adults and had either left the granuloma or been killed within it. To account for the granulomas formed later in the trickle-infected group, we also isolated granulomas from bolus-infected C57Bl/6 mice at 7 days post-infection (early-stage granulomas). To identify the granuloma transcriptional profiles, we extracted the mRNA and quantified transcript levels using the nanostring ‘myeloid innate immunity V2’ panel. For a control, we used naïve mice; as these do not have any granulomas along their small intestine, we harvested intestinal tissue from similar areas to those harvested in infected mice.

**Figure 4:**
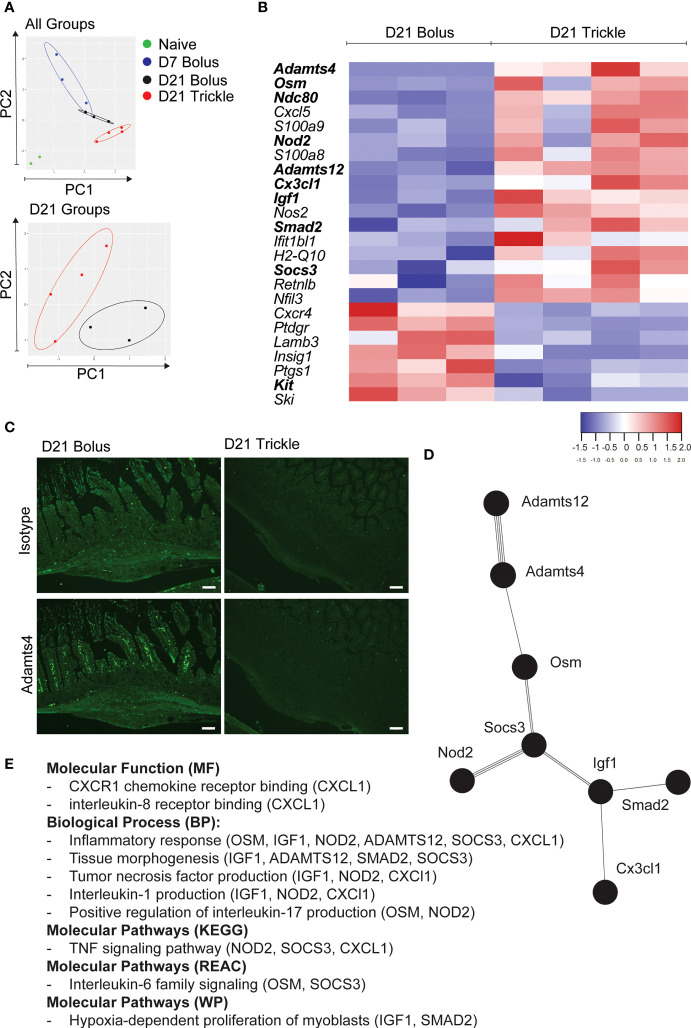
The reduced worm burden in trickle-infected C57Bl/6 mice is associated with an inflammatory and fibrotic transcriptional profile. 6-8 week old C57Bl/6 mice were infected with 200 *H polygyrus* larvae according to the bolus and trickle infection regimes. We used the nanostring nCounter mouse myeloid innate immunity V2 panel to measure the transcription profiles of 754 genes within the granulomas. N=2 for naïve mice, n=3 for D7 and D21 bolus-infected animals and n=4 for trickle-infected mice. **(A)** PCA biplot highlighting gene expression differences between all groups (top) and D21 infected groups (bottom). **(B)** Heatmap showing the relative expression of differentially expressed genes (FC>2) associated with the regulation of myeloid immune responses in D21 trickle *vs*. bolus infected animals. In bold, genes that are also differentially expressed from the ones in granulomas from D7 bolus-infected animals. **(C)** Formalin fixed, paraffin-embedded 6 μm sections were obtained from small intestine swiss rolls. These sections were co-stained with anti-mouse ADAMTS4 or isotype and DAPI. Representative granulomas from bolus- (left) and trickle- (right) infected mice at 21 days post-infection. Antibody stain (bottom), isotype control stain (top). Experiments were performed at least twice (minimum of 2 mice per group) and are representative of a total of 23 (bolus) and 58 (trickle) granulomas. Scale bar = 100μm. **(D)** String analysis (http://string-db.org) of the differentially expressed genes (FC>2) in bold from the list in **(B)** was conducted to obtain the network. **(E)** Gene profiler analysis of molecular function, biological processes and molecular pathways based on gene list from **(D)**.

Using principal component (PC) analysis, we found that the D21 post-trickle infection group clusters separately from any of the other groups (naïve, D7 and/or D21 post bolus-infection, **Figure 4A**). Thirty-eight genes were differentially expressed (DE, adjusted p<0.05 and fold change>2) between naïve intestinal tissue (naive group) and granuloma tissue (infected groups trickle and/or bolus) at day 7 and day 21 post-infection. Ten genes were highly expressed (hDE, adjusted p<0.05 and fold change>16) at all infection time points tested (D7B: D7 bolus, D21B: D21 bolus and D21T: D21 trickle) compared to naïve tissue (**Table 1**, grey rows). All identified hDE genes have already been implicated in the immune response to helminths. Their functions revolve around wound healing (*Chil3, Chil4, Mmp12, Serpine1, Retnla, Arg1*) and the chemoattraction of eosinophils and alternatively activated macrophages (*Rnase2a, Ccl7, Ccl8, Cxcl3*). Of the remaining 23 upregulated genes and five downregulated DE genes, only four (*Cyp1b1*, *Plaur*, *Lamb3* and *Hpgd*) could not be linked to a previous study in helminths. These genes are involved in the alternatively activated macrophage (AAM) phenotype, extracellular matrix (ECM) remodeling and eicosanoid degradation.

**Table 1:** Upregulated genes in all three types of intestinal granulomas (D7 B, D21 B and D21 T, Log2fold>1 in all groups).

	Gene	Function	Nematode Host Response	D21B (BOLUS infection)		D21T (Trickle infection)		D7B (BOLUS infection)	
				Fold Change	Adjusted p-value	Fold Change	Adjustedp-value	Fold Change	Adjusted p-value
**Upregulated in all groups compared to Naive**									
1	*Chil4*	AAM marker Ym2 involved in ECM remodeling	(93)	27756.05	2.14E-18	27070.52	1.06E-18	4384.16	2.17E-12
2	*Rnase2a*	Encodes for eosinophil derived cationic proteins and neurotoxins and involved in neutrophil recruitment	(94)	3400.94	3.42E-13	1864.72	1.46E-11	743.17	9.28E-09
3	*Chil3*	AAM marker Ym1 involved in ECM remodeling	(20, 87, 95)	1886.21	2.82E-48	1868.95	2.17E-50	303.00	1.64E-27
4	*Mmp12*	ECM remodeling	(96)	341.87	1.59E-21	345.71	3.59E-22	29.93	1.66E-07
5	*Serpine1*	Involved in ECM remodeling	(97)	286.56	7.26E-07	151.28	1.29E-05	317.39	5.62E-07
6	*Ccl7*	Chemokine involved in cell migration	(98)	180.60	1.87E-05	259.64	3.42E-06	917.22	1.30E-08
7	*Retnla*	AAM marker, involved in ECM remodeling	(20, 99–101)	123.55	4.20E-65	70.37	6.07E-55	23.52	1.03E-27
8	*Arg1*	Alternatively Activated Macrophage marker involved in ECM remodeling	(7, 16, 35, 87, 102)	86.61	5.17E-45	84.28	8.53E-48	80.52	8.98E-43
9	*Ccl8*	Eosinophil chemotactic factor	(103)	57.52	1.66E-53	68.74	1.58E-62	29.93	3.31E-37
10	*Cxcl3*	Chemokine controlling the adhesion and/or migration of monocytes	(102)	33.53	4.74E-02	41.25	2.86E-02	67.64	1.54E-02
11	*Adam8*	Metalloproteinase, ECM remodeling	(104)	24.23	2.60E-35	21.32	3.94E-33	8.98	3.20E-16
12	*Cxcr4*	Myeloid cell recruitment	(105)	22.27	2.82E-07	4.49	2.00E-02	5.04	1.65E-02
13	*Cd163*	M2 macrophage marker	(106)	14.96	9.12E-07	13.91	4.98E-07	3.92	2.66E-02
14	*Retnlb*	Blocks nematode feeding	(107)	14.78	3.98E-18	31.67	1.40E-32	28.93	8.28E-28
15	*Ccl24*	Eotaxin 2, Eosinophil chemotaxis	(108)	14.62	8.44E-10	32.63	1.28E-17	34.57	2.33E-16
16	*Fn1*	Fibronectin 1, ECM remodeling	(109)	13.63	8.92E-24	9.80	3.26E-20	2.87	1.93E-04
17	*Ccl12*	Chemokine; attracts myeloid cells	(110)	10.48	1.71E-06	15.19	4.54E-09	8.38	2.30E-05
18	*Mrc1*	Mannose Receptor, CD206	(93)	8.92	3.45E-11	7.88	4.84E-11	5.12	1.97E-06
19	*Col1a2*	Collagen I	(111)	8.61	3.02E-84	5.68	1.30E-58	3.03	1.37E-21
**20**	***Cyp1b1***	**Gene involved in the M2 macrophage phenotype**	**No study found**	**7.24**	**4.52E-05**	**6.14**	**1.80E-04**	**9.40**	**4.04E-06**
21	*Mmp19*	Matrix metallopeptidase, breakdown of extracellular matrix	(112)	7.13	1.64E-02	5.55	3.36E-02	8.56	8.32E-03
22	*Pf4*	Chemokine released by platelets that promotes coagulation	(113)	6.24	5.67E-04	7.60	5.88E-05	4.93	3.67E-03
23	*Ccl2*	Monocyte chemoattractant	(114)	6.24	1.01E-04	11.00	4.69E-08	31.38	1.51E-14
24	*Itga5*	Fibronectin receptor alpha	(115)	5.74	7.91E-05	4.87	2.81E-04	3.91	3.61E-03
25	*C3*	Complement component	(116)	5.53	6.31E-16	5.74	2.28E-18	2.76	5.37E-06
26	*Plau*	Urokinase-type plasminogen activator, involved in fibrosis	(117)	5.39	1.82E-10	6.48	1.41E-13	3.37	1.31E-05
27	*Cd68*	Scavenger receptor	(118)	4.67	1.02E-14	4.27	3.00E-14	2.54	1.17E-05
28	*C3ar1*	Complement receptor, inflammatory response	(110)	4.07	3.91E-22	2.74	2.86E-12	2.51	2.31E-09
29	*Col3a1*	Collagen, type III, alpha 1	(119)	3.92	3.84E-09	3.36	4.39E-08	2.00	6.11E-03
30	*Ccl17*	Chemokine, chemoattractant for T-helper cells and Tregs	(120)	3.87	3.65E-03	3.69	3.94E-03	4.83	6.07E-04
31	*Fcgr2b*	IgG receptor	(119)	3.24	2.13E-06	3.90	4.54E-09	2.83	4.22E-05
**32**	***Plaur***	**Plau receptor, involved in the degradation of the ECM**	**No study found**	**2.58**	**3.41E-06**	**2.57**	**1.81E-06**	**2.12**	**4.53E-04**
33	*C5ar1*	Complement 5a receptor, inflammatory response	(121)	2.38	3.59E-05	2.14	2.38E-04	2.28	1.31E-04
	**Downregulated in all groups compared to Naive**								
1	*Nos2*	Inducible Nitric Oxide Synthase involved in Nitric Oxide production	(122)	21.68	6.70E-18	7.42	1.57E-10	10.74	3.52E-12
2	*Enpep*	Glutamyl aminopeptidase	(123)	3.53	2.59E-07	5.72	8.72E-15	2.96	1.48E-05
3	*Crip1*	Involved in fibrosis	(124)	2.79	4.63E-07	3.36	1.33E-10	2.08	5.74E-04
**4**	***Lamb3***	**Regulates cell growth, motility & adhesion. Role in ECM remodeling.**	**No study found**	**2.42**	**4.35E-02**	**9.34**	**8.92E-09**	**5.54**	**3.87E-05**
**5**	***Hpgd***	**Degrades eicosinoids**	**No study found**	**2.37**	**2.46E-03**	**3.57**	**7.50E-07**	**2.18**	**7.51E-03**

At D21 post-infection, 82 upregulated DE genes were identified, of which 26 were highly upregulated hDE genes (grey rows, **Table 2**) compared to genes expressed at D7 post-infection. In the highly upregulated genes in both bolus and trickle-infected animals: seven were associated with mast cells and basophils (*Cpa3, Fcer1a, Ms4a2, Cma1, Tpsb2, Kit, Il3ra*), five with transcriptional regulation (*Msc, Hdac6, Krba1, Smarcd3, Hoxd4*) and two with ECM remodeling (*Trem2, Gata2*). Of the remaining 56 upregulated genes, 17 were involved in wound healing. Eight genes were downregulated in the D21 granulomas compared to the D7 granulomas (*Nox1, Ccl7, Ccl2, Ccl28, Ccl20, Pglyrp1, Ido1, Ptgdr*). These are associated with neutrophil and eosinophil chemoattraction and activation.

**Table 2:** Upregulated genes in intestinal granulomas at 21 days post infection (D21B and D21T, Log2fold>1 in both D21 groups but not in the D7 group).

	Gene	Function	Nematode Host Response	D21 (BOLUS infection)Compared to D7 Bolus		D21 (Trickle infection)Compared to D7 Bolus	
				Fold Change	Adjusted p-value	Fold Change	Adjusted p-value
**Upregulated in both groups compared to D7**							
1	*Cpa3*	Mast cell protease	(125)	3436.54	3.57E-16	3296.97	5.46E-17
2	*Fcer1a*	High affinity Fc epsilon receptor subunit alpha	(126, 127)	1643.11	4.51E-14	1514.01	2.21E-14
3	*Ms4a2*	High affinity Fc epsilon receptor subunit beta	(128)	784.52	5.37E-11	534.85	1.79E-10
4	*Trem2*	ECM remodelling	(129)	324.94	5.63E-10	199.98	7.21E-09
5	*Cma1*	Mast cell chymase gene	(130)	187.22	2.18E-06	133.20	4.27E-06
6	*Igf2*	Hormone similar to insulin (growth factor signalling)	(131)	172.14	9.10E-08	80.95	4.01E-06
7	*Lat*	T cell signalling	(132)	157.93	6.03E-05	75.62	3.57E-04
8	*Flrt2*	Cell adhesion and/or receptor signalling	(109)	93.25	1.54E-05	153.79	5.25E-07
9	*Bcl2*	Regulates cell death	(133)	86.20	3.82E-06	75.53	4.31E-06
10	*Tpsb2*	Serine protease. Found in mast cells	(134)	61.22	7.45E-03	31.98	1.69E-02
**11**	***Krba1***	**Interferes with transcription**	**No study found**	**60.15**	**3.81E-04**	**69.67**	**1.08E-04**
12	*Selp*	Encodes for P-selectin, involved in the recruitment of leukocytes	(135)	47.34	3.76E-04	95.26	7.84E-06
**13**	***Smarcd3***	**Regulates transcription through helicase and ATPase activity**	**No study found**	47.13	2.46E-04	120.87	1.14E-06
14	*Fgf2*	Basic fibroblast growth factor	(136)	46.91	4.19E-04	100.91	6.85E-06
15	*Tlr6*	Innate signalling	(137)	45.69	1.01E-03	67.13	1.21E-04
16	*Gata2*	ECM remodeling	(96)	43.73	1.21E-07	27.11	2.40E-06
17	*Nmb*	Neuropeptide, inhibitor of type-2 inflammation	(138)	42.68	8.41E-03	192.35	4.21E-05
18	*Il3ra*	Key basophil phenotyping marker	(139)	38.93	3.72E-05	31.32	6.64E-05
19	*Kit*	Essential for development and survival of mast cells	(140)	37.61	8.43E-11	18.76	9.65E-08
**20**	***Hoxd4***	**Homeobox genes, involved in morphogenesis and associated with cancer progression**	**No study found**	**37.54**	**1.38E-02**	**33.98**	**9.81E-03**
21	*Hdac6*	Histone deacetylase 6 involved in transcriptional regulation	(141)	29.59	2.23E-03	67.46	4.58E-05
22	*Ccr3*	Chemokine receptor expressed by eosinophils	(142)	24.46	5.06E-04	29.93	8.32E-05
23	*Tlr12*	Innate Signalling	(143)	24.37	2.93E-03	47.75	1.19E-04
24	*Rgs1*	Regulator of G-protein signaling 1, upregulated in IL-4 and Relmalpha stimulated macrophages	(144)	23.73	3.87E-08	27.65	2.31E-09
**25**	***Msc***	**Transcriptional repressor that stimulates Th2/Treg responses**	**No study found**	**23.30**	**2.21E-02**	**28.28**	**8.28E-03**
26	*Cdh13*	Cadherin 13, involved in cell growth, survival and proliferation	(145)	19.68	3.43E-02	56.39	1.20E-03
27	*Cebpg*	Transcription factor, regulates DNA repair	(146)	15.68	2.78E-06	16.59	5.98E-07
**28**	***Adamts12***	**Metalloproteinase**	**No study found**	**15.11**	**2.81E-03**	**109.51**	**1.29E-08**
**29**	***Adamts17***	**Metalloproteinase**	**No study found**	14.80	6.53E-04	31.18	3.28E-06
30	*Gata3*	Transcription factor, Th2 response	(147)	13.43	2.13E-10	17.32	1.66E-13
31	*Ptger2*	Prostaglandin Receptor	(148)	13.36	2.80E-03	18.66	2.85E-04
32	*Alox5*	Lipoxygenase, helps metabolise leukotrienes	(149)	11.93	2.02E-08	14.08	9.29E-11
33	*Mmp12*	*ECM remodeling*	(96)	*11.42*	*4.38E-16*	*11.55*	*1.23E-18*
**34**	***Fgf7***	**Fibroblast Growth Factor**	**No study found**	**10.29**	**4.42E-03**	**15.68**	**2.42E-04**
**35**	***Lif***	**Inhibits Cell Differentiation**	**No study found**	**9.76**	**2.71E-03**	**17.83**	**3.75E-05**
36	*Flt3*	Cytokine receptor, promotes natural helper cell development	(150)	8.52	2.31E-03	8.73	1.02E-03
**37**	***Lamb2***	**Laminin, major component of the basal lamina, associated with collagen networks and regulation of MMPs**	**No study found**	**6.70**	**1.82E-12**	**4.53**	**9.78E-09**
38	*Chil4*	*AAM marker Ym2 involved in wound healing*	(93)	*6.33*	*1.40E-04*	*6.17*	*4.02E-05*
39	*Chil3*	*AAM marker Ym1 involved in wound healing*	(20, 87, 95)	*6.23*	*2.02E-08*	*6.17*	*1.30E-09*
**40**	***Tek***	**Endothelial-specific receptor tyrosine kinase**	**No study found**	**6.07**	**6.54E-06**	**7.41**	**7.08E-08**
41	*Siglecf*	Intestinal eosinophil surface marker	(1)	6.03	4.10E-09	8.63	1.60E-14
**42**	***Adamts2***	**Metalloproteinase**	**No study found**	**5.97**	**3.11E-06**	**5.36**	**2.51E-06**
43	*Itgax*	Integrin, also known as CD11c	(151)	5.90	9.94E-09	5.55	4.81E-09
44	*Retnla*	*AAM marker, wound healing*	(20, 99–101)	*5.25*	*1.02E-12*	*2.99*	*5.07E-07*
45	*Alox15*	Lipoxygenase, helps metabolise leukotrienes	(152)	5.19	2.15E-05	4.00	1.44E-04
46	*Sema5a*	Involved in lymphocyte activation	(153)	5.10	3.37E-04	5.05	1.08E-04
47	*Ccl7*	Chemokine involved in cell migration	(98)	5.08	4.75E-04	3.53	3.24E-03
48	*Ccl2*	Cytokine, also known as MCP-1	(154)	5.03	1.15E-05	2.85	2.27E-03
49	*Ptprb*	Phosphatase, regulates permeability	(109)	4.79	7.92E-07	2.99	3.27E-04
50	*Fn1*	*Fibronectin 1, mesenchymal marker*	(155)	*4.74*	*6.37E-11*	*3.41*	*2.58E-08*
51	*Rnase2a*	*Encodes for eosinophil derived cationic proteins and neurotoxins and involved in neutrophil recruitment*	(94)	*4.58*	*4.10E-06*	*2.51*	*3.26E-03*
52	*Il1r2*	IL-1 decoy receptor	(119)	4.44	2.54E-05	4.00	3.50E-05
53	*Enpp2*	Pyrophosphatase, Also known as autotaxin	(156)	4.15	3.32E-05	3.41	1.85E-04
54	*Il1rl1*	IL-33 receptor	(157)	4.07	8.43E-11	6.81	2.14E-22
55	*Nfatc1*	Transcription factor	(158)	4.02	9.33E-05	2.96	1.52E-03
56	*Alox5ap*	Alox5 activating protein	(119)	4.02	3.17E-16	4.41	3.70E-21
57	*Btk*	Tyrosine kinase	(159)	3.89	6.60E-08	3.45	1.80E-07
**58**	***Cd244***	**Natural Killer Cell receptor involved in cell killing also expressed by eosinophils**	**No study found**	**3.88**	**5.10E-15**	**3.19**	**2.34E-12**
59	*Cd163*	*M2 macrophage marker*	(106)	*3.82*	*7.16E-03*	*3.55*	*4.95E-03*
60	*Hdac5*	Histone deacetylase 5 involved in transcriptional regulation	(141)	3.71	6.87E-09	9.97	7.84E-30
**61**	***Csf2ra***	**Low affinity receptor for CSF2**	**No study found**	**3.56**	**1.12E-02**	**4.39**	**8.96E-04**
62	*Timp3*	Tissue inhibitor of metalloproteinase 3	(160)	3.53	5.51E-17	2.02	9.41E-07
63	*Itgb7*	Integrin beta 7	(161)	3.41	1.91E-02	4.23	1.87E-03
64	*Nfatc2*	Transcription factor	(162)	3.29	5.22E-06	2.13	3.02E-03
65	*Col15a1*	Collagen	(119)	3.26	6.42E-10	3.15	9.29E-11
66	*Vamp2*	Vesicle Associated Membrane Protein 2	**No study found**	3.17	8.85E-06	2.24	9.83E-04
67	*Tgfbr3*	TGF beta receptor III	(163)	3.04	1.23E-06	2.28	1.86E-04
68	*Ctsl*	Cathepsin L, lysosomal peptidase	**No study found**	2.95	4.22E-09	2.70	5.77E-09
69	*Adam19*	Metalloproteinase	(104)	2.80	1.38E-05	2.99	5.38E-07
70	*Mmp13*	Matrix metalloproteinase	(89)	2.77	6.12E-04	4.65	8.03E-09
71	*Igf1*	Hormone similar to insulin (growth factor signalling)	(84)	2.77	1.24E-02	10.58	6.71E-12
72	*Cd34*	Eosinophil adhesion	(164)	2.74	3.83E-07	2.33	4.91E-06
73	*Lat2*	Amino Acid Transporter	(165)	2.73	1.49E-10	2.13	5.34E-07
74	*Adam8*	*Metalloproteinase*	(89)	*2.70*	*4.38E-16*	*2.37*	*7.49E-14*
**75**	***Hpgds***	**Hematopoietic prostaglandin D synthase**	**No study found**	**2.67**	**2.30E-05**	**4.49**	**5.23E-13**
**76**	***Vav1***	**Vav1 1 oncogene**	**No study found**	**2.57**	**2.13E-05**	**2.47**	**1.04E-05**
77	*Tnc*	Extracellular matrix glycoproteins	(166)	2.48	4.22E-09	3.97	5.08E-23
78	*Pbx1*	Pre B cell leukemia homeobox 1	(167)	2.41	2.09E-05	2.60	5.06E-07
79	*Icosl*	ICOS ligand	(112)	2.36	3.64E-04	2.13	7.90E-04
80	*Ctgf*	Connective tissue growth factor	(168)	2.30	2.58E-02	3.00	8.27E-04
**81**	***S100a4***	**S100 calcium binding protein A4**	**No study found**	**2.28**	**5.96E-04**	**2.98**	**4.28E-07**
82	*C3*	Complement component	(116)	2.01	4.31E-04	2.08	4.93E-05
**Downregulated in both groups compared to D7**							
1	*Nox1*	Codes for NADPH oxidase 1	(168)	10.39	2.38E-05	6.46	2.13E-04
2	*Ccl2*	Cytokine, also known as MCP-1	(154)	5.03	1.15E-05	2.85	2.27E-03
3	*Ccl7*	*Chemokine involved in cell migration*	(98)	*5.08*	*4.75E-04*	*3.53*	*3.24E-03*
4	*Ccl28*	Increases the migration of CCR3 expressing eosinophils	(169)	3.26	4.22E-04	4.61	5.83E-07
5	*Ccl20*	Implicated in the formation and function of mucosal lymphoid tissues	(168)	3.12	9.80E-04	6.06	1.31E-08
6	*Ido1*	involved in tryptophan metabolism	(170)	2.34	9.30E-03	2.15	9.81E-03
**7**	***Pglyrp1***	**Pro-inflammatory innate immunity protein found in the granules of eosinophils and macrophages**	**No study found**	**2.16**	**1.83E-08**	**2.36**	**8.51E-12**
8	*Ptgdr*	Prostaglandin receptor, involved in the chemotaxis of leukocytes (e.g. eosinophils)	(171)	2.02	7.49E-05	9.14	1.63E-25

When looking at differential expression between trickle- and bolus-infected granuloma tissue, we also found striking differences (**Figure 4B**; **Table 3**). At D21 post-infection, 17 genes were upregulated in trickle- *vs*. bolus-infected groups, three of which were hDE. The 2 most upregulated genes were *Adamts4* (~x124) and *Osm* (~x49). They were not upregulated in bolus-infected animals on either D7 or D21 compared to naïve intestinal tissue (fold change<2 and/or p>0.05). ADAMTS proteins, a type of matrix metalloproteinase (MMPs), have been implicated in tissue scarring (63). Interestingly, despite a dramatic increase in *Adamts4* expression in the trickle-infected animals (~x124, **Table 3**), protein levels were increased in bolus but not trickle-infected animals (**Figure 4C**).

**Table 3:** Differentially expressed genes between D21B and D21T groups (Log2fold>1 in both groups).

	Gene	Function	Nematode Host Response	D21 BOLUS vs. TRICKLE infection		D21 BOLUS infection VS. NAIVE		D21 TRICKLE infection vs NAIVE		D7 BOLUS infection vs NAIVE	
				Fold Change	Adjusted p-value	Fold Change	Adjusted p-value	Fold Change	Adjusted p-value	Fold Change	Adjusted p-value
**Genes upregulated with trickle infection**												
**1**	*Adamts4*	**Metalloproteinase** **(ECM remodelling)**	**No study found**	**123.96**	**7.87E-05**	**<2**	**>0.05**	**94.57**	**1.32E-03**	**2.88**	**>0.05**
**2**	*Osm*	**Secreted cytokine Growth regulator** **(cytokine/growth factor signalling)**	**No study found**	**49.22**	**1.67E-02**	**<2**	**>0.05**	**73.15**	**1.48E-02**	**<2**	**>0.05**
3	*Ndc80*	Kinetochore complex component, regulated by IL-4 in resident macrophages (cell cycle & apoptosis)	(172)	22.04	1.07E-03	-11.18	2.07E-02	<2	4.87E-01	-58.52	6.79E-04
4	*Cxcl5*	*Chemokine produced by eosinophils*	(173)	*12.56*	*9.85E-04*	*13.53*	*>0.05*	*169.92*	*9.56E-05*	*137.52*	*2.82E-04*
5	*S100a9*	*Controls macrophage accumulation & cytokine production*	(174)	*12.13*	*3.50E-04*	- <2	*>0.05*	*12.63*	*1.55E-03*	*22.21*	*1.60E-04*
6	*Nod2*	Pattern Recognition Receptor	(175)	8.06	2.42E-03	-8.76	2.91E-03	<2	>0.05	-11.57	1.46E-03
7	*S100a8*	*Controls macrophage accumulation & cytokine production*	(174)	*7.82*	*1.15E-02*	<2	*>0.05*	*6.86*	*3.06E-02*	*12.94*	*4.49E-03*
**8**	*Adamts12*	***Metalloproteinase***	***No study found***	***7.25***	***4.65E-06***	***-3.72***	***1.17E-02***	***<2***	***>0.05***	***-56.23***	***7.66E-06***
9	*Cx3cl1*	Fraktalkine, involved in fibrosis	(176)	5.44	1.10E-08	-4.51	1.32E-05	<2	>0.05	-3.61	2.82E-04
10	*Igf1*	*Hormone similar to insulin* *Shales the macrophage activation phenotype*	(84)	*3.82*	*6.07E-05*	*-* <2	*>0.05*	*3.54*	*7.99E-04*	*-2.99*	*2.01E-02*
11	*Nos2*	Inducible Nitric Oxide Synthase involved in Nitric Oxide production	(177)	2.92	5.63E-03	-21.68	6.70E-18	-7.42	1.57E-10	-10.77	3.52E-12
12	*Smad2*	Signal transducer for the TGFbeta receptor	(178)	2.39	5.50E-06	-3.11	9.41E-08	- <2	>0.05	-4.44	3.57E-12
13	*Ifit1bl1*	*IFN‐stimulated genes*	(179)	*2.34*	*1.02E-03*	*-5.52*	*2.71E-10*	*-2.36*	*1.54E-03*	***-* <**2	*>0.05*
**14**	*H2-Q10*	***MHC I gene***	***No study found***	***2.19***	***1.56E-03***	<2	***>0.05***	***3.67***	***1.14E-06***	***5.11***	***4.21E-09***
15	*Socs3*	Tumour suppressor, limiting intestinal epithelial cell proliferation	(180)	2.17	4.43E-03	-2.25	7.16E-03	- <2	>0.05	-2.11	1.60E-02
16	*Retnlb*	Blocks nematode feeding	(181)	2.14	8.05E-03	14.78	3.98E-18	31.67	1.40E-32	28.93	8.28E-28
17	*Nfil3*	Non classical regulatory T cell gene	(182)	2.08	4.95E-03	<2	>0.05	2.76	2.40E-04	**<2**	>0.05
**Genes downregulated with trickle infection**												
1	*Cxcr4*	*Eosinophil recruitment during Type-2 granuloma formation*	(181)	*4.96*	*5.68E-04*	*22.27*	*2.82E-07*	*4.49*	*2.00E-02*	*5.04*	*1.65E-02*
2	*Ptgdr*	*Binding by prostaglandin D2 activates eosinophils*	(183)	*4.54*	*7.90E-11*	*2.42*	*9.91E-04*	<2	*>0.05*	*4.87*	*1.73E-10*
**3**	***Lamb3***	***Regulates cell growth, motility & adhesion. Role in wound healing.***	***No study found***	***3.86***	***5.94E-04***	***-2.42***	***4.35E-02***	***-9.34***	***8.92E-09***	***-5.54***	***3.87E-05***
**4**	***Insig1***	**Regulates cholesterol biosynthesis**	**No study found**	**2.13**	**9.96E-05**	**2.19**	**4.31E-04**	**<2**	**9.41E-01**	**3.50**	**6.57E-09**
5	*Ptgs1*	Macrophage polarisation in presence of IL-4	(184)	2.05	9.51E-07	2.61	3.72E-08	<2	2.56E-01	<2	>0.05
6	*Kit*	*Essential for development and survival of mast cells*	(140)	*2.01*	*4.93E-02*	*5.86*	*2.40E-05*	*2.92*	*1.50E-02*	*-6.42*	*6.36E-03*
7	*Ski*	TGFβ signalling	(185)	2.00	5.01E-07	2.77	9.88E-10	<2	>0.05	2.27	2.08E-06

Unsurprisingly, the gene expression profile of infected animals was skewed towards fibrosis and scarring with increased *Col1a* (seen in all granulomas studied, gene 19 **Table 1**), increased *Tnc* (tenacin, increased in D21 *vs*. D7 granulomas, gene 77 **Table 2**) and late deposition of *Fn1* (fibronectin, increased in D21 *vs*. D7 granulomas, gene 50 **Table 2**) (64). A key difference in ECM content in tissue scarring *vs*. tissue regenerative healing includes low MMP : TIMP ratio (64). This ratio was low in all granulomas but more so in the trickle D21 granulomas: *Mmp12* and *Mmp13* are increased in both types of D21 granulomas (genes 33 and 70 **Table 2**), while *Timp3* is increased 1.75-fold in trickle *vs*. bolus D21 granulomas (**Supplementary Table 1**).

In bolus-infected animals, the granulomas collected at D21 were all of similar ‘age’, generated at the same time in response to one large dose of larvae. In contrast, in the trickle-infected animals, granulomas collected at D21 will have a range of ‘ages’ ranging from 10 to 21 days. To assess whether the differences observed could be attributed to time, we tested whether the differences between the D21 trickle and bolus granulomas were also observed when comparing D7 *vs*. D21 granulomas. Seven genes were identified (*Cxcl5, S100a9, S100a8, H2-q10, Ifit1bl1, Cxcr4* and *Kit*) where timing likely had an impact. The fold changes for the D21T granulomas fall either between the D7 and D21 bolus granulomas or close to the D7 granuloma values. These genes are involved in events linked to early granuloma functions like cell migration/accumulation and inflammation, or mast cells which are present in late-stage rather than early-stage granulomas. However, 9 genes (*Adamts4, Osm, Ndc80, Igf1, Nod2, Adamts12, Smad2, Socs3* and *Cx3cl1*) are significantly different between D21T *vs.* D7 and D21T *vs.* D21B. As such, the difference observed is likely not due to the difference in ‘age’ of the granulomas but can be attributed to the infection regimen (in bold, **Figure 4B**). String analysis using these 9 genes generated a pathway linking 8 of the genes (**Figure 4D**). A functional analysis of this pathway using g:Profiler, identified inflammation, tissue morphogenesis and myoblast proliferation as key functions (**Figure 4E**; **Supplementary Table 2**).

By contrast, at day 21 post-infection, only seven genes were downregulated in D21T *vs*. D21B. Of the seven, one was also downregulated between D21T *vs*. D7B (grey row in **Table 3**, *Kit*) and four were downregulated between D21B *vs.* D7B (in italics in **Table 3**, *Cxcr4, Ptgdr, Lamb3* and *Kit*). Two genes were not associated with a helminth study (in bold in **Table 3**, *Lamb3* and *Insig1*).

## Discussion

Resistance to *H. polygyrus* bolus infections is strain dependent with the C57Bl/6 strain being susceptible to *H. polygyrus* infections and carrying high burden chronic infections. This contrasts with the BALB/c mice which have lower worm burdens and are considered resilient to infection (13). We and others (13, 65) have shown that *H. polygyrus*-infected BALB/c mice have more granulomas, as well as higher levels of Th2 cytokines and parasite specific IgG1 antibodies compared to susceptible C57Bl/6 mice. These differences contribute to the BALB/c resilient phenotype (13). Despite the BALB/c strain’s resilience to *H. polygyrus*, we found no differences in BALB/c mice between the two infection models (trickle/bolus), which agrees with findings from *Trichuris muris* trickle *vs*. bolus nematode infection studies (57). Therefore timings/dose may have less of an impact in naturally resilient animals compared to more susceptible individuals.

Trickle infections, which mimic the grazing behavior of livestock and continual exposure of humans in endemic areas, result in decreased worm burdens in laboratory animals (40, 41, 62). Using our trickle model, C57Bl/6 mice had fewer worms than those infected with a single bolus infection. Resilience to a bolus *H. polygyrus* infection has been attributed to a strong Th2 response, requiring tuft cells activation, Th2 cytokine production (IL-4 and 13 in particular), the formation of granulomas, IgG1 production, and the activation of alternatively activated macrophages and mast cells (3, 66). Other changes in intestinal physiology and immune markers have also been observed but not directly linked to resilience (3, 66). In our model, despite the reduced parasite numbers in trickle-infected animals, we were surprised that many of the resilience-associated parameters were unchanged, especially since previous studies using *H. polygyrus* trickle infection models have linked resilience to increased *Il4* expression levels in the MLN and increased serum IgG1 (41, 67).

Our data show that resilience in the trickle-infected C57Bl/6 mice was not associated with increased Th2 cytokine levels (IL-4, 5, 9, 10 and 13) measured in the intestinal content, MLN and/or SPL. We saw no difference in many of the genes associated with typical Th2 anti-*H. polygyrus* responses. Expression of ECM remodeling genes (*Chil4, Chil3, Mmp12, Serpine 1, Retlna*, and *Arg1*), myeloid cell recruitment genes (*Ccl7, Ccl8 Cxcl3, Cxcr4, Ccl24, Ccl12 and Ccl2)* and genes with direct antiparasitic activity (*Retlnb*) was similar between all infected groups tested (D7, D21B and D21T).

Goblet cells are responsible for the production and release of mucins that protect the intestinal epithelium against pathogens. During helminth infections, IL-4 and IL-13 stimulate goblet cell hyperplasia, increasing mucin secretion which helps expel intestinal worms (68–71). We found an increase in goblet cells and intestinal scrapings weight (composed mainly of mucus) in both bolus- and trickle-infected mice, with no difference in the *Muc2/Muc5a* gene expression ratio. Our results differ from work using *Trichuris* trickle-infected mice, where resistance was associated with increased IL-13 and its effector mechanisms including goblet cell hyperplasia and Muc5ac production (57). This difference could be accounted for by the different parasite lifecycles. While they are both enteric, *T. muris* lives in the caecum and does not fully reside within the intestinal tissue while *H. polygyrus* can be found in the small intestine either within the tissue or coiled around villi. IL-4 and IL-13 (27), and as such *H. polygyrus* infection (55), also promote intestinal smooth muscle contraction, thought to promote worm expulsion. Trickle-infected animals had faster intestinal transit times despite lower levels of intestinal IL-4. The decrease in transit time was also observed in bolus-infected animals at D7, where levels of IL-4 were also low, indicating that the effect may be time dependent rather than due to the mode of infection.

Mast cell deficient mice have reduced alarmin (IL-25, IL-33 and TSLP) and Th2 cytokine expression, as well as increased worm burdens (72). Mast cells have also been linked to reduced *H. polygyrus* fecundity (73). Four of the top five upregulated genes in the D21 (B and T) *vs*. D7 granuloma areas were genes linked to mast cells (cpa3, *Fcer1a*, *Ms4a2 and* Cma1). Since the granulomas used for our studies at D21 did not contain worm larvae, the mast cells present in these late-stage granuloma areas may be participating in the wound healing process. Mast cells have previously been implicated in both cellular and molecular mechanisms of wound healing (reviewed in (74)). Surprisingly, differences in serum IgE levels between bolus and trickle-infected animals were not correlated to differences in *Fcer1a* gene expression (receptor for the IgE antibody). Interestingly, *kit*, an essential mast cell developmental and survival factor, is downregulated in the D21T compared to D21B granuloma areas. Since its expression levels are between the levels measured at D7 and D21B, this may be a function of time from granuloma formation rather than an inherent difference between the modes of infection.

Resistant strains of mice develop faster and more intense parasite specific antibody responses following *H. polygyrus* infections, as compared to susceptible strains, where isotypes IgG_1_, IgA and IgE have been linked to worm clearance (3, 37, 75). Passive transfer of serum, and specifically IgG_1_, from infected mice results in decreased adult worm burden and fecundity (67, 76–78). *H. polygyrus* adult numbers are increased in infected mice lacking IgA (79). IgG_1_ and IgE have also been negatively correlated with worm survival across different strains of mice (80–82). We found no differences in IgG1 serum or intestinal IgA levels, and the small increases in serum IgE observed are unlikely to solely account for the worm burden differences between the groups. This again is surprising since others have related increased levels of serum IgG1 to resistance in *H. polygyrus* trickle infected mice albeit at later time points (41, 67). Eliminating tissue stage parasites is thought to rely on antibody dependent cell mediated cytotoxicity (ADCC) by macrophages and eosinophils (7, 15, 16), the main cellular players within the granuloma. Antibodies are key to this process and we observed the presence of IgG1 in the parasite-containing granulomas of the trickle compared to bolus-infected mice which could account for the improved clearance.

Despite many aspects of the host responses being similar between our two modes of infection, we did observe two significant differences: an increase in tuft cell number and granuloma number in the trickle-infected groups. The tuft cells could be generating a faster response to the incoming larvae, also indicated by the presence of IgG1 around developing worms in the trickle-infected granulomas, leading to more granuloma formation and effective killing of the parasites. Time rather than magnitude of the response may be at play here, which would also explain why all worms in the trickle-infected animals were not cleared. The earlier doses could have avoided the effective anti-tissue dwelling parasite responses that develop over time in the trickle infected animals.

Granulomas eventually disappear once the worms have been killed and/or escaped into the lumen (53). Many studies have focused on understanding the mechanisms involved in damaging/killing worms, but fewer have focused on the wound healing processes involved in creating and resorbing a granuloma. This is key, as there is a delicate balance between parasite clearance and host pathology, which ultimately impacts host fitness. Since trickle-infected mice encounter worms over a period of time compared to the one-time dose in bolus-infected mice, we were particularly interested in the impact of trickle infection on the balance of parasite killing *vs.* tissue regeneration and scarring in the intestinal tissue. Hyperproliferation/Hyperplasia of intestinal smooth muscle cells leads to a lasting thickening of the intestinal wall. Increases in IGF1, accompanied by altered levels of SOCS3, have been associated with smooth muscle hyperplasia in the context of Crohn’s disease (83). While we saw no differences in the density of collagen deposition or the expression of collagen genes, we did observe an increase in the area of smooth muscle within the granulomas in the trickle-infected animals, associated with increased levels of *Igf1*, *Socs3*, *Osm, Nod2* and *Cx3cl1* in the granuloma areas and a generally more inflammatory profile. In *Nippostrongylus brasiliensis* infection, IGF1 promotes worm expulsion and acute wound healing. Animals lacking *Igf1* had higher worm counts than their wildtype counterparts (84) and IGF1 was associated with acute wound healing and reduced lung damage/hemorrhage during early infection (52). The impact of IGF1 in a more chronic setting or in the context of the small intestine during a helminth infection has yet to be assessed. OSM has been associated with the accumulation of profibrotic macrophages (85). NOD2 KO animals have impaired healing in the skin (86) and in the gut (87). Elevated *Nod2* levels in our trickle-infected animals may be due to the ongoing tissue damage that the intestinal mucosa is undergoing with the entry/exit of larval worms spread over time. Increased *Cx3cl1* in granulomas has recently been associated with increased levels of Hyaluronic acid (HA) during schistosomiasis (88). HA is a marker of tissue scarring, strengthening the trickle-infected animals’ strong ‘scarring’ phenotype.

To the best of our knowledge, we are the first to implicate the ADAMTS family of proteins (extra cellular matrix degrading enzymes) during helminth infections. Increased levels of the expression of ADAM proteins (*Adam8* and *Adam19*), related to the ADAMTS family, have been measured in *Fasciola*-infected sheep livers (89). We identified increases in *Adamts2, 4, 12* and *17* within D21 granulomas with levels of *Adamts4* and *12* being increased in trickle-infected animals. We attempted to correlate scarring to ADAMTS levels, the most highly differentially expressed ADAMTS protein family member in our granuloma areas. However, protein levels did not match the transcriptional profile observed: bolus-infected animals had elevated levels of ADAMTS while trickle-infected animals did not. ADAMTS protein regulation is complex and not yet fully understood (90). We are therefore unable to tell whether the lack of ADAMTS4 in the trickle-infected animals is due to an initial lack of protein or to high protein turn-over. Levels of AGGRECAN protein expression and *Versican* gene expression, both ADAMTS4 targets, were not detectable in the intestine (data not shown). ADAMTS4 has also been shown to increase tumour growth (91), which correlates with the increased size of the granulomas from trickle-infected animals. More recently, ADAMTS4 was also found to promote chronic airway inflammation by supporting immune cell infiltration at the expense of lung function (92), which correlates with the more inflammatory profile of the granulomas from trickle-infected animals. Further study on the ADAMTS family and its role during granuloma formation/resorption is needed. To fully understand the function of the ADAMTS family, single cell RNA sequencing profiles of early/late-stage granulomas with/without encysted worms and late-stage scar tissue from trickle/bolus-infected mice would provide a more detailed view of the cellular processes underlying the host response to parasitic worms in more natural contexts. This would allow the identification of mechanisms which regulate granuloma formation/resorption and their impact on parasite and host fitness.

Our study highlights the complexities of host-parasite interactions. To study the delicate balance between tissue damage and parasite clearance, we need to use a range of outputs (not just worm burden and systemic Th2 responses) in models which better mimic natural conditions such as trickle infections, re-infections, and co-infections.

## Data availability statement

The datasets presented in this study can be found in online repositories. The names of the repository/repositories and accession number(s) can be found in the article/**Supplementary Material**.

## Ethics statement

The animal study was reviewed and approved by University of Calgary’s Life and Environmental Sciences Animal Care Committee (protocol AC17-0083) and the University of California, Riverside’s Institutional Animal Care and Use Committee (https://or.ucr.edu/ori/committees/iacuc.aspx; protocol A-20180023).

## Author contributions

Study conception and design: AA, SYK, MGN, and CAMF; data collection: AA, SYK, HL, WNTN, SP, ALC, MCL, JB, ES, and CAMF; analysis and interpretation of results: AA, SYK, HL, SP, SMJP, KDP and CAMF; draft manuscript preparation: AA, SYK, SMJP, JDW, MGN, and CAMF. All authors contributed to the article and approved the submitted version.
