# Corrigendum: Trickle infection with *Heligmosomoides polygyrus* results in decreased worm burdens but increased intestinal inflammation and scarring

**DOI:** 10.3389/fimmu.2024.1432056

**Published:** 2024-06-11

**Authors:** Anupama Ariyaratne, Sang Yong Kim, Stephen M. J. Pollo, Shashini Perera, Hongrui Liu, William N. T. Nguyen, Aralia Leon Coria, Mayara de Cassia Luzzi, Joel Bowron, Edina K. Szabo, Kamala D. Patel, James D. Wasmuth, Meera G. Nair, Constance A. M. Finney

**Affiliations:** ^1^ Department of Biological Sciences, Faculty of Science, University of Calgary, Calgary, AB, Canada; ^2^ Host Parasite Interactions Training Network, University of Calgary, Calgary, AB, Canada; ^3^ Division of Biomedical Sciences, School of Medicine, University of California Riverside, Riverside, CA, United States; ^4^ Faculty of Veterinary Medicine, University of Calgary, Calgary, AB, Canada; ^5^ Departments of Physiology and Pharmacology, Faculty of Medicine, University of Calgary, Calgary, AB, Canada

**Keywords:** helminth, granuloma, trickle infection, ADAMTS, intestinal parasite, tissue scarring

## Error in Figure/Table

In the published article, there was an error in **Figure 1** as published. The original numbers used for the C56Bl/6 data in panel C were incorrect by a factor of 5. The corrected **Figure 1** and its caption ‘The reduced worm burden in trickle-infected C57Bl/6 mice is associated with elevated serum IgE’ appear below.

**Figure 1:**
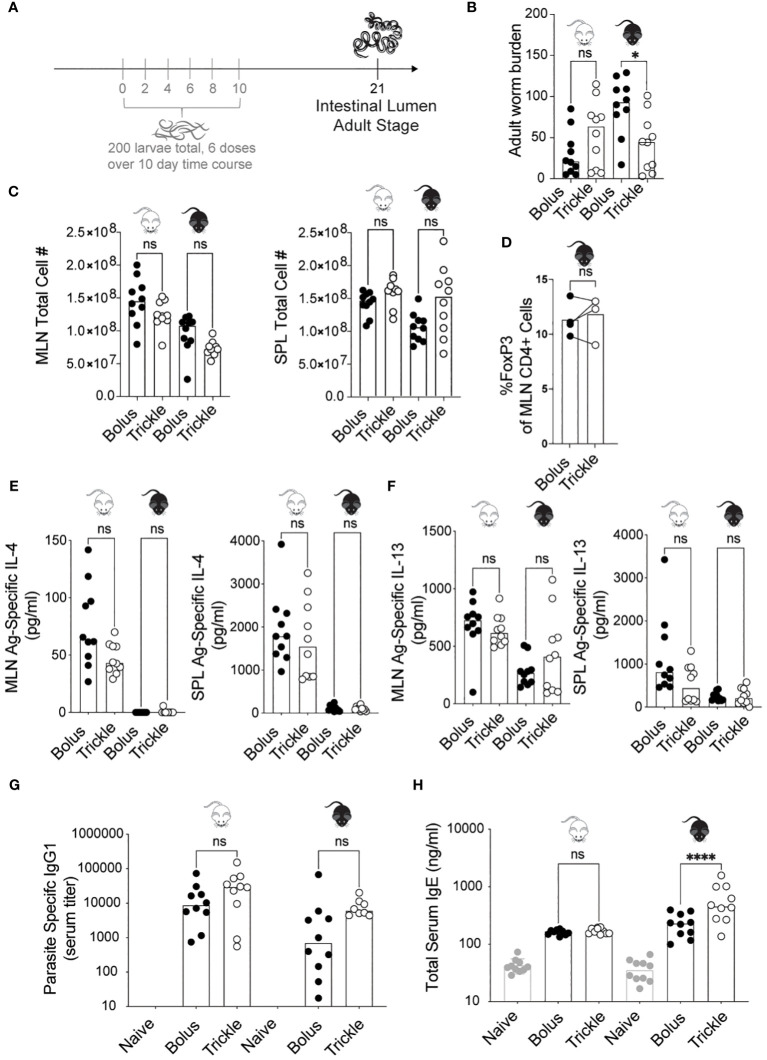
The reduced worm burden in trickle-infected C57Bl/6 mice is associated with elevated serum IgE. 6-8 week old C57Bl/6 and BALB/c mice were infected with 200 *H polygyrus* larvae according to the bolus and trickle infection regimes. **(A)** Trickle infection regime: mice are infected with 200 larvae in total, but in multiple doses over the course of infection (in grey). Doses of ~33 larvae are trickled on days 0, 2, 4, 6, 8 and 10 post-infection. There is a 10-day window after the final dose to allow parasites to fully develop into adults and migrate from the intestinal tissue to the intestinal lumen. **(B)** Adult worms were counted in the small intestine using a dissection microscope. **(C)** Single cell suspensions were isolated from the mesenteric lymph nodes and spleens. Viable cell numbers in the MLN (left) and SPL (right). Single cell suspensions were either used for flow cytometry or cultured for 48 hours in the presence of *H polygyrus* antigen. **(D)** Percentage of Foxp3^+^ cells within the CD4+ population of MLN cells were measured by flow cytometry. **(E)** IL-4 (MLN and SPL) and **(F)** IL-13 (MLN and SPL) cytokine levels were measured in the supernatant by ELISA. Serum antibody levels were measured by ELISA for **(G)** parasite specific IgG1 and **(H)** total IgE. Levels in naïve controls were undetectable for parasite specific IgG1. For all panels except D, graphs represent pooled data from 2 experiments, bars represent the median, with a minimum of 3 mice per group per experiment. For panel D, each circle represents one experiment using cells pooled from 5 mice. BALB/c mice (white mouse) and C57Bl/6 mice (black mouse) were infected according to the bolus (black circles) and trickle (white circles) regimes. A normality test was performed (Anderson-Darling) followed by a Kruskal Wallis test with Dunn’s multiple comparisons test to test for statistical significance between trickle and bolus groups; for panel **(D)**, a paired T-test was performed; n.s., not significant; *p<0.05, ****p<0.0001.

The authors apologize for this error and state that this does not change the scientific conclusions of the article in any way. The original article has been updated.

## Text correction

In the published article, there was an error. In the results section, reference was made to differences in cell numbers between C57Bl/6 and Balb/c mice. This was incorrect.

A correction has been made to the Results section entitled ‘Increased worm clearance in trickle-infected animals is associated with increased levels of serum IgE, but no other changes in key systemic cytokine or antibody responses, paragraph 2. This sentence previously stated:

‘Despite BALB/c mice having considerably higher cell numbers than C57Bl/6 mice at both time points in both organs, we found no differences in cell number between the trickle- and bolus-infected groups in either of the organs (**Figure 1C**).”

The corrected sentence appears below:

‘We found no differences in cell number between the trickle- and bolus-infected groups in either of the organs (**Figure 1C**).”

The authors apologize for this error and state that this does not change the scientific conclusions of the article in any way. The original article has been updated.

